# Isolate-Specific Modulation of Growth, Carbon Allocation, and Transcriptomic Responses in *Spirodela polyrhiza* by Duckweed-Associated Bacteria

**DOI:** 10.3390/microorganisms14081850

**Published:** 2026-08-20

**Authors:** Karnjana Ruenpham, Kazuhiro Mori, Yasuhiro Tanaka, Arinthip Thamchaipenet, Masaaki Morikawa, Tadashi Toyama

**Affiliations:** 1 Interdisciplinary Graduate School of Medicine and Engineering, University of Yamanashi, 4-3-11, Takeda, Kofu 400-8511, Yamanashi, Japan; k.ruenpham@gmail.com; 2 Graduate Faculty of Interdisciplinary Research, University of Yamanashi, 4-3-11, Takeda, Kofu 400-8511, Yamanashi, Japan; mori@yamanashi.ac.jp (K.M.); yasuhiro@yamanashi.ac.jp (Y.T.); 3 Department of Genetics, Faculty of Science, Kasetsart University, Bangkok 10900, Thailand; fsciatt@ku.ac.th; 4 Duckweed Holobiont Resource & Research Center (DHbRC), Kasetsart University, Bangkok 10900, Thailand; morikawa@ees.hokudai.ac.jp; 5 Division of Biosphere Science, Graduate School of Environmental Science, Hokkaido University, Sapporo 060-0810, Hokkaido, Japan

**Keywords:** growth-promoting bacteria, starch accumulation, transcriptome

## Abstract

Duckweed-associated plant growth-promoting bacteria have attracted attention for their potential to enhance duckweed biomass production. However, the mechanisms underlying isolate-specific growth promotion are unclear. This study compared the effects of two duckweed-associated bacterial isolates, *Terrimicrobium* sp. PS02 and *Aeromicrobium* sp. PS05, on the growth, biomass composition, colonization behavior, and transcriptomic responses of *Spirodela polyrhiza.* Biomass composition and transcriptome analyses were performed to characterize the host responses. Both isolates enhanced duckweed biomass; PS02 increased it 1.2-fold, whereas PS05 induced a significant 1.4-fold increase compared to the uninoculated control. PS05 established bacterial populations approximately one order of magnitude higher than those of PS02 and formed dense extracellular polymeric substance-mediated microcolonies on the surface. Transcriptome analysis revealed that PS05 induced 1108 differentially expressed genes, compared with 454 in PS02, indicating greater host transcriptomic reprogramming. Functional enrichment of transcriptome responses showed that PS05 preferentially regulates carbohydrate biosynthesis, central carbon metabolism, and starch biosynthesis, significantly enhancing starch accumulation and turion formation without reducing protein or photosynthetic pigment content. This study provides evidence linking bacterial colonization, host transcriptomic regulation, carbon allocation, and biomass production in duckweed, offering a basis for engineering high-performance duckweed–microbiome systems for sustainable biomass production.

## 1. Introduction

Duckweeds (*Lemnaceae*) are the smallest and fastest-growing flowering aquatic plants. Owing to their rapid biomass production, high protein and starch content, and ability to efficiently assimilate nutrients from water, duckweeds have attracted increasing attention as sustainable biomass resources for biofuels, animal feed, fertilizers, bioplastics, and wastewater treatment [1,2,3]. Furthermore, duckweeds can be cultivated without competing for agricultural land and can efficiently use nutrients recovered from wastewater, thereby contributing to resource recycling and sustainable circular bioeconomy systems [4,5,6]. Moreover, cultivation in controlled indoor environments enables year-round production of hygienically safe biomass with stable quality and predictable productivity, independent of seasonal fluctuations [7,8].

Duckweed growth and productivity are strongly influenced by microorganisms inhabiting plant surfaces and the surrounding aquatic environment. Similar to terrestrial plants, duckweeds harbor diverse plant growth-promoting bacteria (PGPB) that enhance host growth through mechanisms such as indole-3-acetic acid (IAA) production, siderophore-mediated iron acquisition, nitrogen fixation, phosphate solubilization, and stress alleviation [9,10,11,12]. Several duckweed-associated bacteria, including *Acinetobacter soli* W7-21, *Aquitalea magnusonii* H3, *Exiguobacterium* sp. MH3, *Acidobacteriota*-related bacteria F-183 and TBR-22, and other aquatic PGPB have been reported to promote duckweed growth and biomass production [9,11,13,14]. These studies highlight the importance of plant–microbe interactions in duckweed biotechnology.

Microbiome studies have demonstrated that duckweeds harbor taxonomically distinct and host-specific bacterial communities, suggesting that the associated microorganisms play important roles in host growth and adaptation. Furthermore, bacterial colonization mechanisms and host–microbe interactions have been investigated using genomic and microbiome approaches [15,16,17,18]. These findings support the concept that duckweeds are holobionts consisting of a host plant and its associated microbiota.

Despite these advances, the mechanisms underlying the isolate-specific effects of duckweed-associated bacteria remain unclear. Most previous studies have focused on the isolation and screening of beneficial bacteria or the evaluation of their growth-promoting effects [9,12,14,19]. Although some studies have compared multiple bacterial isolates and examined their colonization of duckweed surfaces, their analyses have been largely limited to growth responses, biomass accumulation, and chlorophyll production. Consequently, the mechanisms by which different bacterial isolates differentially influence host physiology and metabolism remain unclear, particularly regarding the association between differences in bacterial colonization strategies and host growth responses, biomass composition, or molecular regulation in duckweed. To the best of current knowledge, no study has systematically compared the effects of different duckweed-associated bacterial isolates on the same host plant by integrating bacterial colonization patterns, host biomass productivity, biochemical composition, and global transcriptomic responses. Understanding such isolate-specific interactions is important for elucidating the ecological basis of duckweed–bacteria symbiosis and developing rational strategies for selecting beneficial microbial partners for high-performance duckweed cultivation systems.

This study hypothesized that different duckweed-associated bacteria establish distinct symbiotic relationships with their hosts, consequently inducing different physiological and molecular responses. To test this hypothesis, this study aimed to investigate the isolate-specific effects of two bacterial isolates, *Terrimicrobium* sp. PS02 and *Aeromicrobium* sp. PS05, isolated from *Spirodela polyrhiza*. The present study characterized the plant growth-promoting traits of these bacteria, evaluated their colonization patterns on duckweed surfaces, and assessed their effects on duckweed growth, biomass composition, and host transcriptomic responses. This study provides the first integrated comparison of how different duckweed-associated bacteria modulate host growth, metabolism, and gene expression, thereby advancing the understanding of isolate-specific duckweed–bacteria symbiosis and providing a scientific basis for the development of optimized duckweed–microbiome systems for sustainable biomass production.

## 2. Materials and Methods

### 2.1. Duckweed and Its Cultivation

*Spirodela polyrhiza* UY001, originally collected from Kofu, Yamanashi, Japan, was maintained under sterile conditions in 0.1× diluted Hutner’s medium (0.1 DHM) [20]. Duckweed cultures were incubated in a plant growth chamber at 28 ± 1 °C under fluorescent lamps at a photosynthetic photon flux density of 150 µmol m^−2^ s^−1^ and a 16 h photoperiod (16 h light/8 h dark), and sub-cultured every 1–2 weeks. Axenic conditions were routinely verified by placing duckweeds on Reasoner’s 2A (R2A; Shiotani M.S., Amagasaki, Japan) agar and confirming the absence of bacterial growth after 7 days of incubation.

### 2.2. PGPB Isolates

Two duckweed-associated PGPB isolates, *Terrimicrobium* sp. PS02 and *Aeromicrobium* sp. PS05, were previously isolated from the surface of *S. polyrhiza* cultivated in environmental pond water containing indigenous bacterial communities. Briefly, duckweed fronds were vigorously vortexed, shaken, and vortexed again in sterile 0.1 DHM to detach surface-associated bacteria. The resulting suspension was serially diluted and spread onto R2A agar plates, followed by incubation at 25 °C for 14 d. Representative colonies were isolated, and genomic DNA was extracted using Cica Geneus DNA Extraction Reagent (Kanto Chemical, Tokyo, Japan) according to the manufacturer’s instructions. Full-length 16S rRNA gene amplification and sequencing were conducted by FASMAC (Atsugi, Japan). The resulting sequences were deposited in the DNA Data Bank of Japan under accession numbers LC943439 and LC943440. Taxonomic identification was conducted using the web-based NCBI BLAST analysis of the 16S rRNA gene sequences against type strains, and plant growth-promoting traits, including IAA and siderophore production and potential nitrogen fixation, were evaluated as described in Table S1.

### 2.3. Co-Cultivation of S. polyrhiza and Each PGPB

Each PGPB isolate was grown in R2A broth at 150 rpm and 28 °C for 24 h, centrifuged at 10,000× *g* for 5 min, washed two times with sterile 0.1 DHM, and resuspended in sterile 0.1 DHM. A bacterial cell suspension with an optical density of 0.2 at 600 nm was prepared to a final volume of 100 mL in a 300 mL flask. A total of 30 fronds of axenic *S. polyrhiza* were inoculated with each bacterial suspension and incubated for 24 h under the cultivation conditions described above. Subsequently, 20 inoculated fronds (average dry weight 4.4 ± 0.6 mg) were gently washed three times using sterile 0.1 DHM and transferred into a 500 mL flask containing 200 mL of 0.1 DHM in three biological replicates, alongside uninoculated axenic duckweed controls. All flasks were maintained under identical conditions for 10 d. At the end of the co-cultivation period, the duckweed biomass was harvested. A portion of the biomass was dried (70 °C for 24 h), while the remaining fresh biomass was collected for further analyses.

### 2.4. Bacterial Cell Density Measurement

To quantify bacterial colonization on the duckweed surface, the colony-forming units (CFU) on the fronds and roots were counted 24 h post-inoculation and on the final day of co-cultivation. Plant bodies with two fronds were washed two times with sterile water, sectioned using a scalpel to separate the fronds and root tissues, and the total number of roots was counted. Frond and root tissues were separately suspended in sterile 0.1 DHM, serially diluted to 10^−4^, and 10 µL aliquots of each dilution were spotted onto R2A agar plates. All plates were incubated at 28 °C until visible bacterial colonies appeared. The number of colonies was counted and used to calculate the CFU per frond and root, factoring in the initial suspension volume, the respective dilution multipliers, and the 10 µL plating volume. This bacterial cell density quantification was performed using three biological replicates.

### 2.5. Scanning Electron Microscopy

Duckweed–PGPB co-cultivation samples were observed using a scanning electron microscope (SEM) on day 3. Duckweed samples were submerged in 2% glutaraldehyde in 0.1 M phosphoric acid buffer (pH 7.4). The samples were analyzed by Filgen, Inc. (Nagoya, Japan), and the bacterial cells attached to the frond and root surfaces were visualized using a JEOL SEM instrument (JSM-7500F; JEOL, Tokyo, Japan).

### 2.6. Biochemical Analysis of Duckweed Biomass

The effect of PGPB on duckweed growth was evaluated by assessing the pigment, starch, and protein content, along with the overall biomass enhancement. Pigments were extracted from fresh duckweed on the final day of co-cultivation. Two duckweed fronds (average fresh weight 10.2 ± 2.6 mg) were placed into a tube containing 1 mL of pre-chilled 99.5% methanol and incubated for 40 min on ice in the dark. The absorbance was measured at 470, 652, and 665 nm using a UV-Vis spectrophotometer (Shimadzu, Tokyo, Japan). The total chlorophyll and carotenoid contents were calculated using standard equations described by [21].

Subsequently, the dried biomass was ground into a powder and measured to determine the total starch and protein contents. Starch content was measured using a total starch assay kit (Megazyme International, Wicklow, Ireland) according to the manufacturer’s instructions. Protein content was quantified using a bicinchoninic acid protein assay kit (Takara Bio Inc., Otsu, Shiga, Japan) following the manufacturer’s instructions. All biochemical analyses were conducted using three biological replicates. In addition, to check for turion formation in duckweed, fresh samples on the last day of co-cultivation were observed using a stereomicroscope (Olympus SZ61; Olympus, Tokyo, Japan).

### 2.7. RNA Extraction, Sequencing, and RT-qPCR

Total RNA was extracted from control *S. polyrhiza* and duckweed–PGPB co-cultivated samples (PS02 and PS05) on day 10 using NucleoSpin RNA Plant (MACHEREY-NAGEL, Düren, Germany) following the manufacturer’s instructions with three biological replicates per treatment. Library preparation, RNA sequencing, and differential expression analyses were performed by FASMAC (Atsugi, Japan). The sequences were deposited in the Gene Expression Omnibus under accession number GSE338012. The *S. polyrhiza* 162 (SP162) was used as the reference genome for read mapping [22]. Principal component analysis (PCA) and pairwise Pearson correlation analysis were performed using log2-transformed transcripts per million (log_2_(TPM + 1)) values. Differentially expressed genes (DEGs) were defined using an adjusted *p*-value < 0.05 and absolute log_2_(fold change) ≥ 0.58. RT-qPCR was performed to validate the RNA sequencing data. *Actin* was used as a stable reference gene, and all primer sequences are listed in Table S2. Extracted RNA was subjected to RT-qPCR across three biological replicates using the One-step TB Green PrimeScript RT-PCR kit and a Thermal Cycler Dice Real-Time System II (Takara Bio Inc., Otsu, Japan) with the following thermal cycling conditions: 42 °C for 5 min; 95 °C for 10 s; 40 cycles of 95 °C for 5 s and 60 °C for 30 s. Relative gene expression was quantified using the 2^−ΔΔCt^ method [23] and expressed as the log-transformed fold change (log_2_FC).

### 2.8. Functional Annotation and Pathway Enrichment

A custom annotation pipeline was used to elucidate the putative functions of DEGs. Gene Ontology (GO) terms were assigned using EggNOG-mapper v2 against the eggNOG database v5 in DIAMOND mode [24,25]. Kyoto Encyclopedia of Genes and Genomes (KEGG) orthology terms were independently assigned using KofamKOALA [26] and merged with the corresponding annotations retrieved from the EggNOG-mapper. Functional enrichment of DEGs was performed using the R package clusterProfiler to identify transcriptomic changes associated with duckweed–PGPB interactions.

### 2.9. Statistical Analyses

All statistical analyses were conducted using R version 4.6.0 (R Foundation for Statistical Computing, Vienna, Austria) and RStudio (Posit Software, PBC, Boston, MA, USA). One-way analysis of variance, followed by Tukey’s post hoc test, was performed to determine the statistical significance of dry biomass, starch, protein, and pigment content in duckweed biomass. In addition, independent two-sample Welch’s *t*-tests were performed to evaluate differences in bacterial colonization between the two PGPB isolates. Statistical significance was set at *p* < 0.05.

## 3. Results

### 3.1. Isolate-Specific Growth Promotion and Colonization Patterns of Duckweed-Associated PGPB

**Figure 1:**
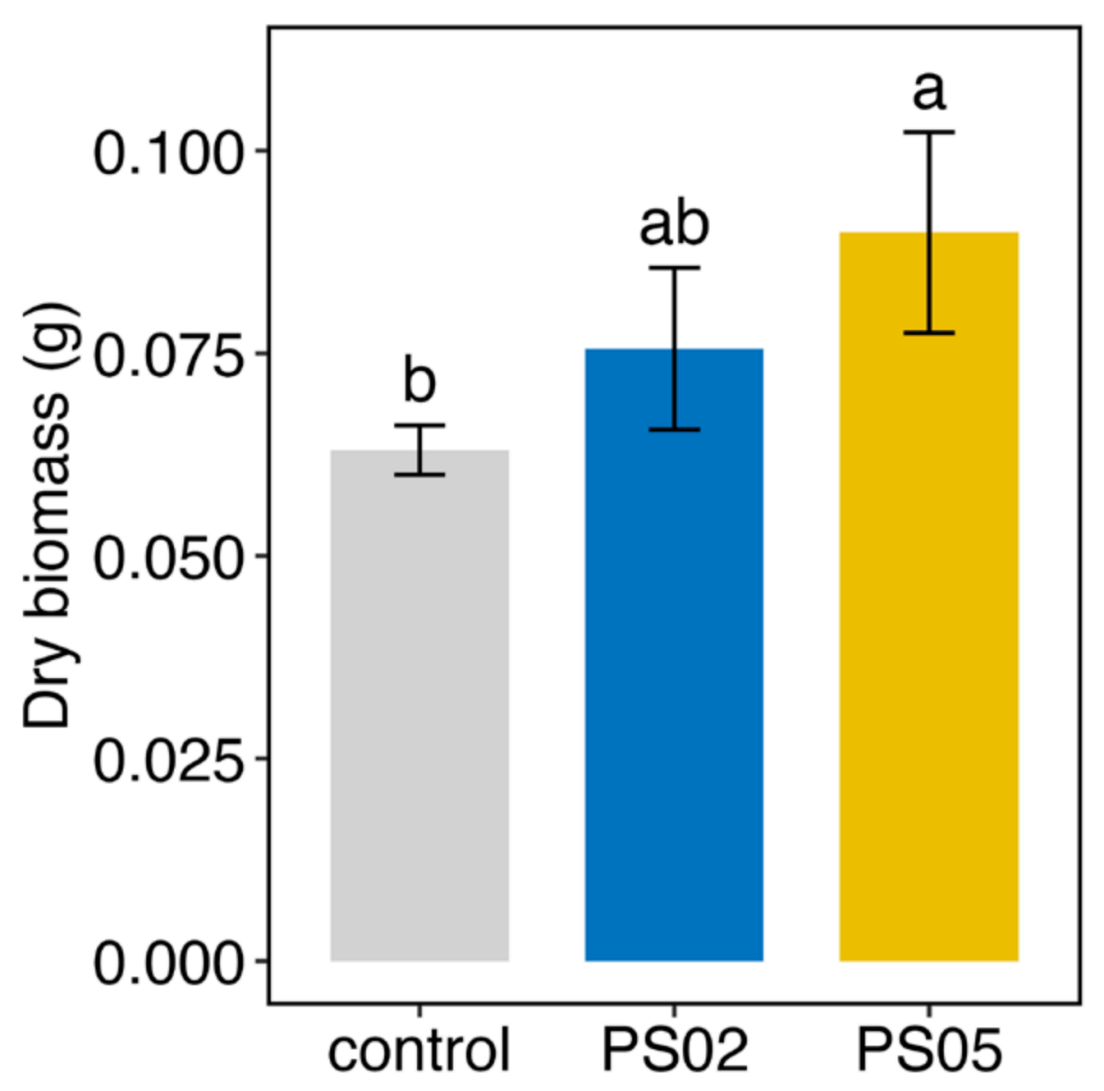
Isolate-specific promotion of *Spirodela polyrhiza* dry biomass after 10 d of co-cultivation with duckweed-associated plant growth-promoting bacteria (PGPB). Axenic duckweed (control) was co-cultivated with *Terrimicrobium* sp. PS02 and *Aeromicrobium* sp. PS05 for 10 d. Error bars represent the standard deviation of the biological replicates, and different letters above the bars indicate significant differences at *p* < 0.05, using one-way analysis of variance, followed by Tukey’s post hoc test.

Two duckweed-associated PGPB isolates, PS02 and PS05, were individually co-cultured with axenic *S. polyrhiza* for 10 d to compare their isolate-specific growth-promoting effects. Although both isolates tended to increase duckweed biomass (Figure 1), only PS05 induced a statistically significant increase compared to the control (*p* < 0.05). The final biomass reached 0.0899 g dry weight in the PS05 treatment (a 1.4-fold increase), whereas the PS02 treatment resulted in a dry weight of 0.0756 g (a 1.2-fold increase). PS05 exhibited a greater growth-promoting effect than that of PS02.

**Figure 2:**
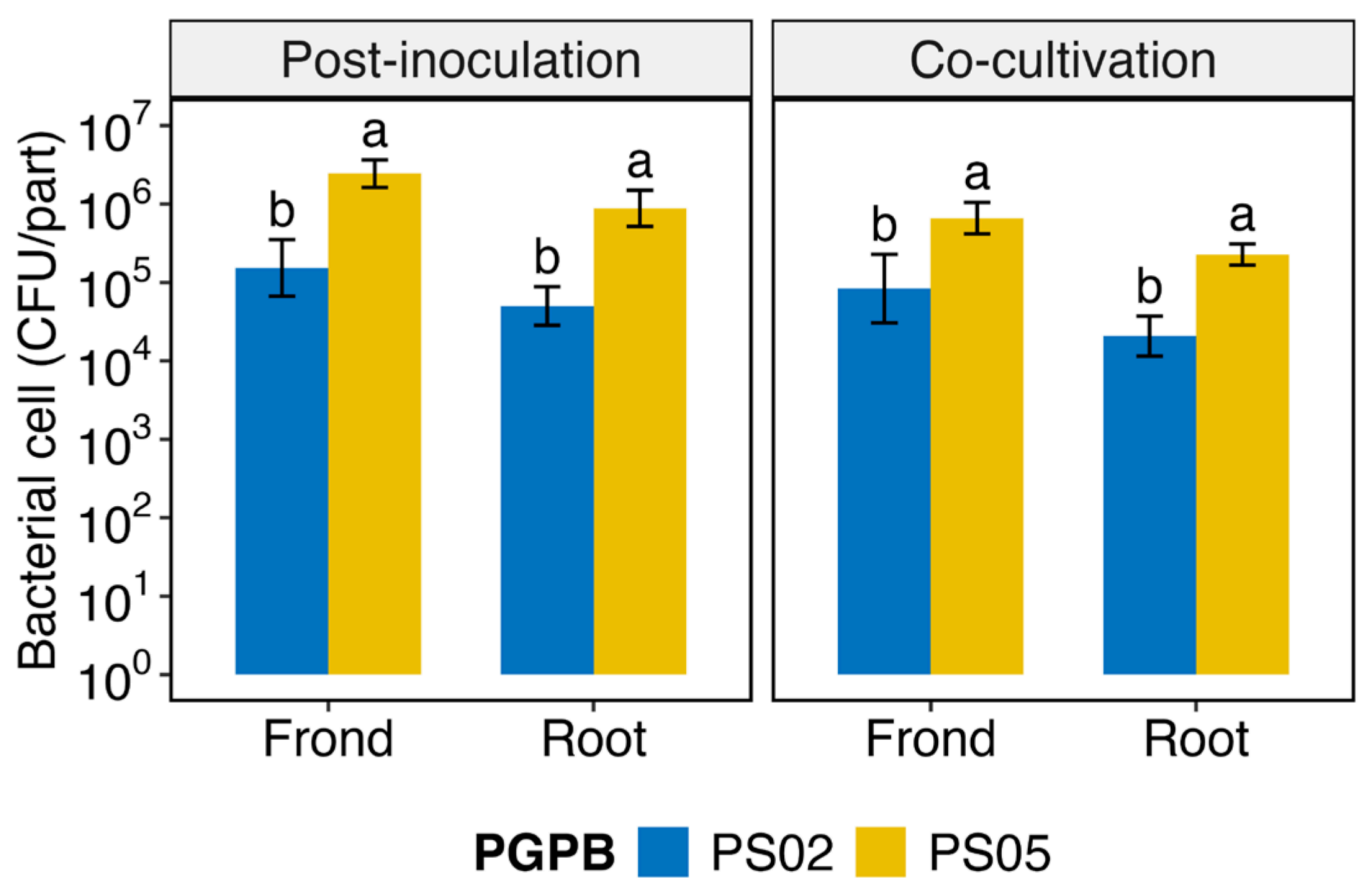
Bacterial colonization dynamics on *Spirodela polyrhiza* fronds and roots. Bacterial cell density was quantified 24 h post-inoculation and after 10 d of co-cultivation with duckweed-associated PGPB. The blue bars represent *Terrimicrobium* sp. PS02, and the yellow bars represent *Aeromicrobium* sp. PS05. Error bars represent the standard deviation of the biological replicates, and different letters above the bars indicate significant differences between the two bacterial isolates within each tissue type and time point, as determined by independent two-sample Welch’s *t*-tests (*p* < 0.05).

Both isolates successfully colonized the duckweed surface but exhibited markedly different colonization dynamics (Figure 2). After 24 h, PS05 reached bacterial densities of 2.43 × 10^6^ CFU/frond and 8.80 × 10^5^ CFU/root, whereas PS02 reached only 1.53 × 10^5^ CFU/frond and 5.00 × 10^4^ CFU/root, representing approximately 16-fold and 18-fold lower densities, respectively. After 10 d of cultivation, the bacterial populations decreased on the fronds but remained relatively stable on the root. Nevertheless, PS05 consistently maintained substantially higher cell densities (6.60 × 10^5^ CFU/frond and 2.27 × 10^5^ CFU/root) than PS02 on both fronds and roots (8.33 × 10^4^ CFU/frond and 2.07 × 10^4^ CFU/root).

**Figure 3:**
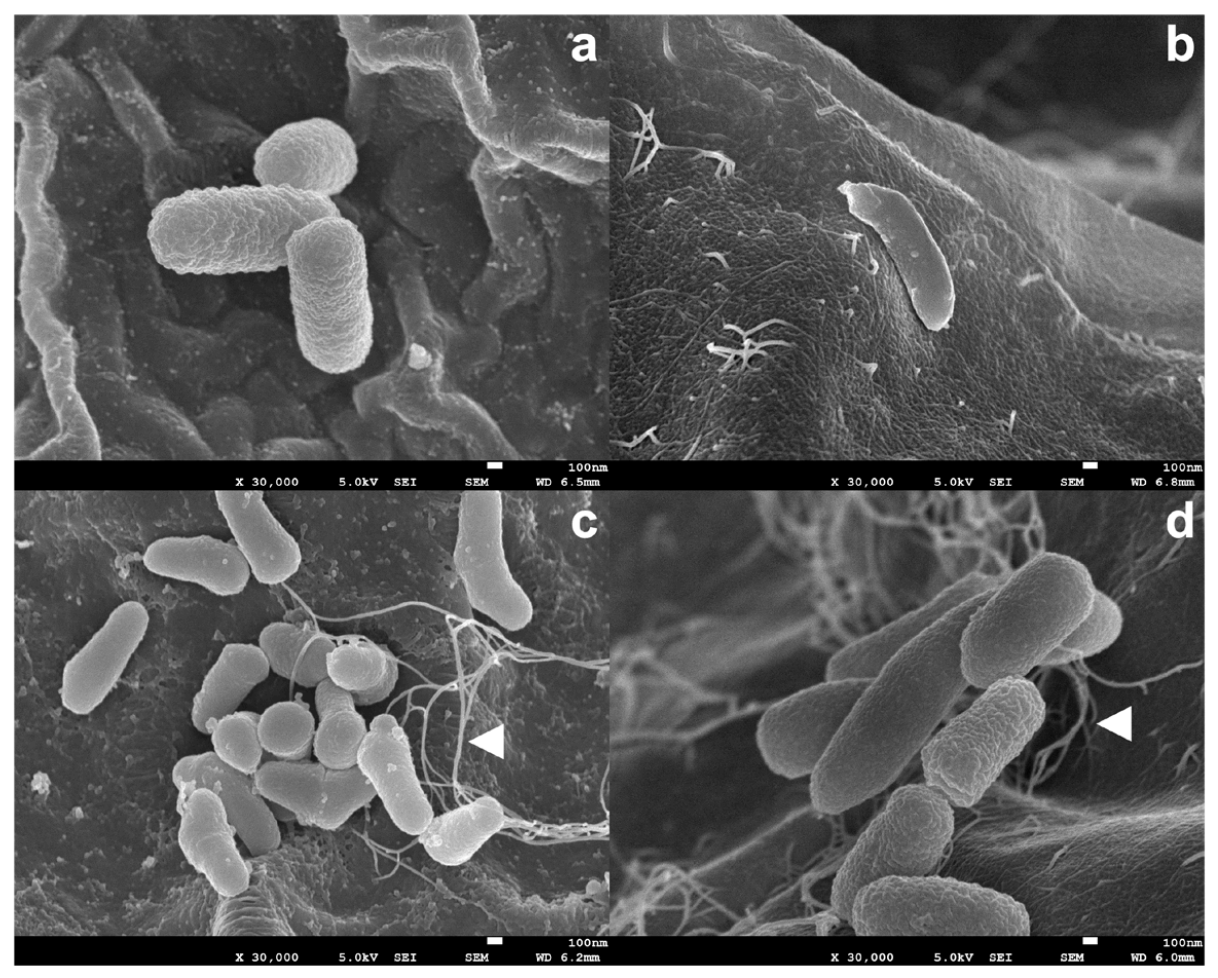
Scanning electron microscopy (SEM) images of *Spirodela polyrhiza* surfaces on day 3 of co-cultivation with duckweed-associated PGPB. All images were captured at a magnification of 5000×. (**a**,**b**) *Terrimicrobium* sp. PS02 attached to the frond (**a**) and root (**b**) surfaces. (**c**,**d**) Microcolony formation and extracellular polymeric substance (EPS)-mediated attachment of *Aeromicrobium* sp. PS05 to frond (**c**) and root (**d**) surfaces. White arrowheads indicate filamentous extracellular polymeric substances.

The contrasting colonization patterns were further confirmed using SEM analysis (Figure 3). PS02 cells were sparsely distributed on the frond and root surfaces and were mainly observed as individual cells or small aggregates consisting of one to three cells (Figure 3a,b). In contrast, PS05 formed dense microcolonies composed of approximately 10–20 cells interconnected by abundant filamentous extracellular polymeric substances (EPS), resulting in extensive attachment across the plant tissues (Figure 3c,d).

### 3.2. Isolate-Specific Effects of Two PGPB Isolates on Biomass Composition of S. polyrhiza

**Figure 4:**
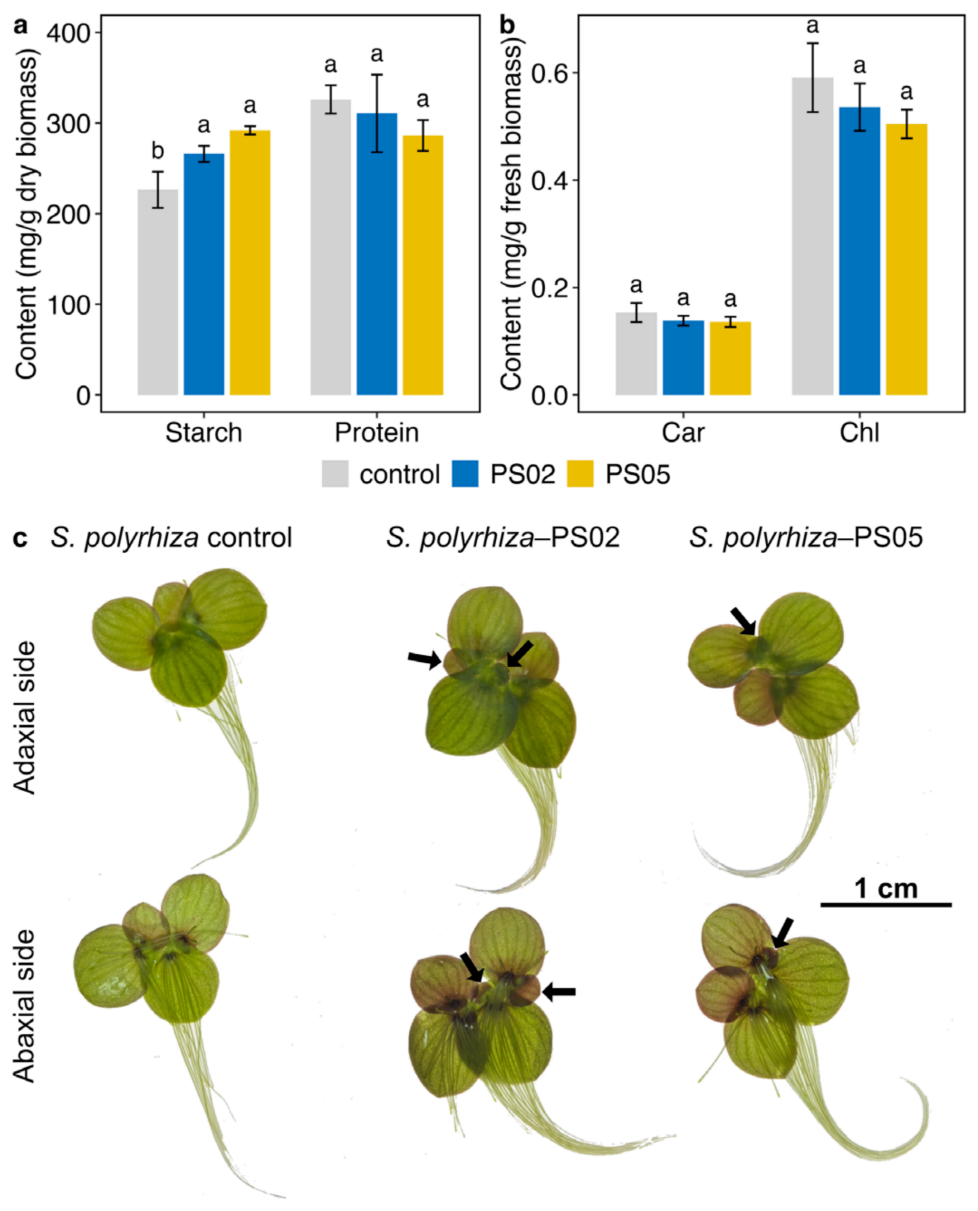
Effect of duckweed-associated PGPB on *Spirodela polyrhiza* biochemical composition and morphology following 10 d of co-cultivation. (**a**) Starch and protein content (mg/g dry biomass). (**b**) Photosynthetic pigment content, including total carotenoid (Car) and total chlorophyll (Chl) (mg/g fresh biomass). The gray bars represent axenic controls, blue bars represent *Terrimicrobium* sp. PS02, and yellow bars represent *Aeromicrobium* sp. PS05. Error bars indicate the standard deviation, and different letters above the bars indicate significant differences using one-way analysis of variance, followed by Tukey’s HSD post hoc test (*p* < 0.05). (**c**) Representative stereomicroscopic images of *S. polyrhiza* grown under axenic conditions (control) or co-cultivated with PGPB isolates. The top row displays the adaxial (upper) surface, and the bottom row displays the abaxial (lower) surface. Black arrows indicate the initial formation of turions on the adaxial and abaxial surfaces of the mother frond induced by both PGPB isolates. Scale bar = 1 cm.

After 10 d of co-cultivation, the biochemical composition of *S. polyrhiza* biomass was evaluated (Figure 4). Among the biomass components analyzed, starch was the only component statistically significantly enhanced by bacterial inoculation (*p* < 0.05; Figure 4a). The starch content of axenic *S. polyrhiza* was 226 ± 19.9 mg/g dry biomass, whereas co-cultivation with PS02 and PS05 increased the starch content to 266 ± 8.89 mg/g (1.2-fold) and 292 ± 4.58 mg/g (1.3-fold), respectively (*p* < 0.05). This increase was greater in duckweed co-cultivated with PS05 than that in duckweed co-cultivated with PS02.

In contrast, the protein content was not significantly affected by either bacterial isolate (*p* > 0.05; Figure 4a). The protein content was 326 ± 15.5, 311 ± 42.8, and 286 ± 17.0 mg/g dry biomass in the control, PS02, and PS05 treatments, respectively. Similarly, bacterial inoculation had little effect on the photosynthetic pigment content (Figure 4b). The total carotenoid content remained between 0.14 and 0.15 mg/g fresh biomass, whereas the total chlorophyll content ranged from 0.50 to 0.59 mg/g fresh biomass, with no significant differences among treatments *(p* > 0.05).

Stereomicroscopy revealed distinct morphological responses to bacterial inoculation (Figure 4c). Axenic duckweed remained vegetative throughout the cultivation period, whereas duckweed co-cultivated with either PS02 or PS05 consistently initiated visible turion formation on the fronds, particularly on the abaxial surface.

### 3.3. Transcriptomic Responses of S. polyrhiza to Duckweed-Associated PGPB

RNA sequencing was performed using three biological replicates of axenic *S. polyrhiza* (control) and duckweed co-cultivated with PGPB isolates PS02 and PS05. An average of 43.9 million clean reads was obtained for each sample. High sequencing quality was achieved across all libraries, with Q30 values exceeding 90.8% and GC content exceeding 56.7% (Table 1).

**Figure 5:**
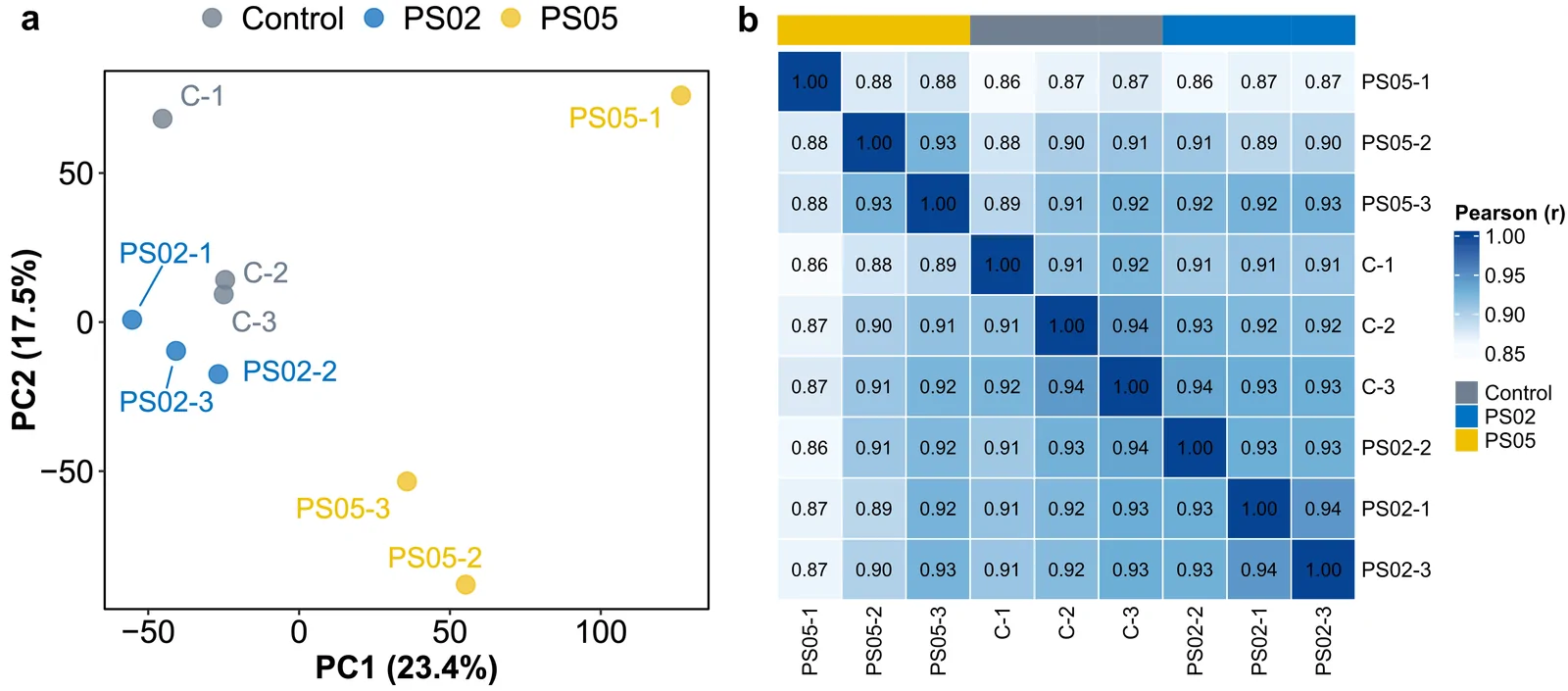
Principal component analysis (PCA) and correlation heatmap of duckweed–PGPB co-cultivation transcriptomic profiles. (**a**) PCA was generated using log_2_-transformed transcripts per million (log_2_(TPM + 1)) values. (**b**) Pairwise Pearson correlation analysis was performed using log_2_(TPM + 1) values. Gray represents the axenic *S. polyrhiza* control; blue represents *S. polyrhiza* co-cultivated with *Terrimicrobium* sp. PS02, and yellow represents *S. polyrhiza* co-cultivated with *Aeromicrobium* sp. PS05. The samples are shown as independent biological replicates.

To compare the global transcriptomic responses among the treatments, principal component analysis (PCA) was performed using log_2_-transformed transcripts per million (log_2_(TPM + 1)) values (Figure 5a). The control and PS02-treated samples clustered closely, indicating similar global gene expression profiles. In contrast, the PS05-treated samples were clearly separated from the control and PS02 groups along PC1, demonstrating a distinct transcriptomic response.

The pairwise Pearson correlation analysis further supported these PCA results (Figure 5b). The expression profiles of the control and PS02 samples were highly similar (*r* ≥ 0.91), whereas the correlations between the PS05-treated samples and the other treatments were lower (*r* = 0.86–0.92). These results indicate that PS05 induced substantially greater transcriptomic changes in *S. polyrhiza* than PS02. This greater transcriptomic reprogramming aligns with the superior growth-promoting effects observed in PS05-treated duckweed.

### 3.4. Differential Gene Expression Induced by Duckweed-Associated PGPB

**Figure 6:**
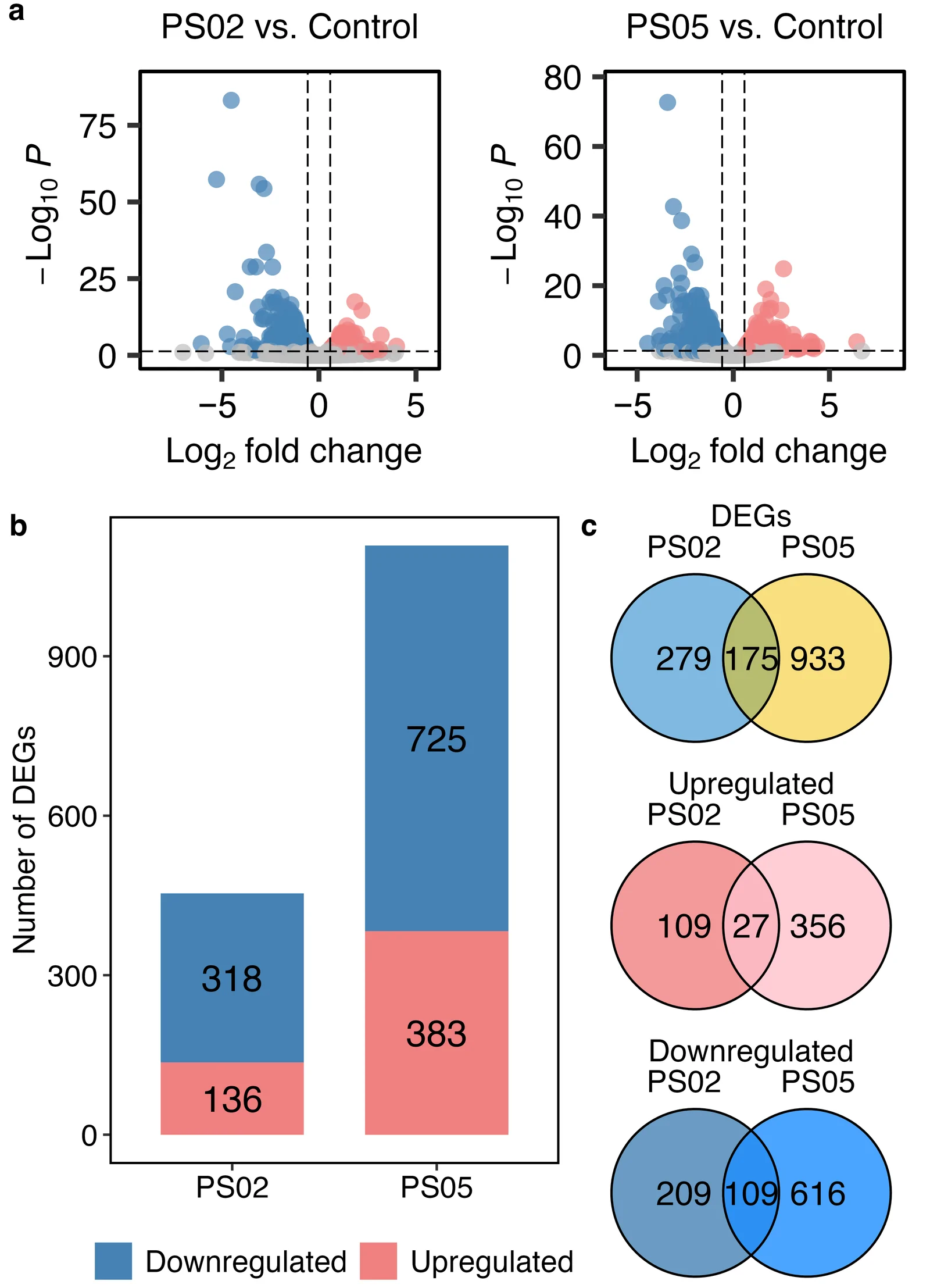
Comparative transcriptomic analysis of *Spirodela polyrhiza* following 10 d of co-cultivation with PGPB. (**a**) Volcano plots illustrating the distribution of differentially expressed genes (DEGs) induced by *Terrimicrobium* sp. PS02 and *Aeromicrobium* sp. PS05 relative to axenic control. The *x*-axis represents the log_2_(fold change), and the *y*-axis represents –log10(adjusted *p*-value). (**b**) Stacked bar charts quantifying the total number of upregulated and downregulated DEGs. (**c**) Venn diagrams showing the shared and unique DEGs induced by the two distinct PGPB isolates. Across all panels, red and blue represent upregulated and downregulated genes, respectively.

To investigate the host molecular responses to duckweed-associated PGPB, DEGs were identified using an adjusted *p*-value < 0.05 and |log_2_(fold change)| ≥ 0.58 (1.5-fold change). Co-cultivation with PS02 resulted in 454 DEGs, comprising 136 upregulated and 318 downregulated genes (Figure 6a,b). In contrast, PS05 induced substantially larger transcriptomic changes, with 1108 DEGs, including 383 upregulated and 725 downregulated genes. Thus, PS05 affected approximately 2.4 times more genes than PS02.

Both bacterial isolates predominantly induced transcriptional repression, as the number of downregulated genes exceeded the number of upregulated genes in both treatments (Figure 6b). Comparative analysis further revealed highly isolate-specific transcriptional responses (Figure 6c). Although 175 DEGs were shared between the two treatments, PS05 induced 933 unique DEGs, indicating a substantially broader transcriptional response than PS02.

### 3.5. Functional Enrichment of Differentially Expressed Genes

GO enrichment analysis was performed to identify the biological processes affected by the bacterial inoculation (Table 2). Five significantly enriched biological process terms were identified in PS02-treated duckweed, whereas three were identified in PS05-treated duckweed.

PS02 primarily affected stress- and defense-related processes, including secondary metabolic processes and responses to salicylic acid and water deprivation. In contrast, PS05 significantly enriched carbohydrate biosynthetic, phenylpropanoid metabolic, and secondary metabolic processes, indicating marked changes in host carbon metabolism.

**Figure 7:**
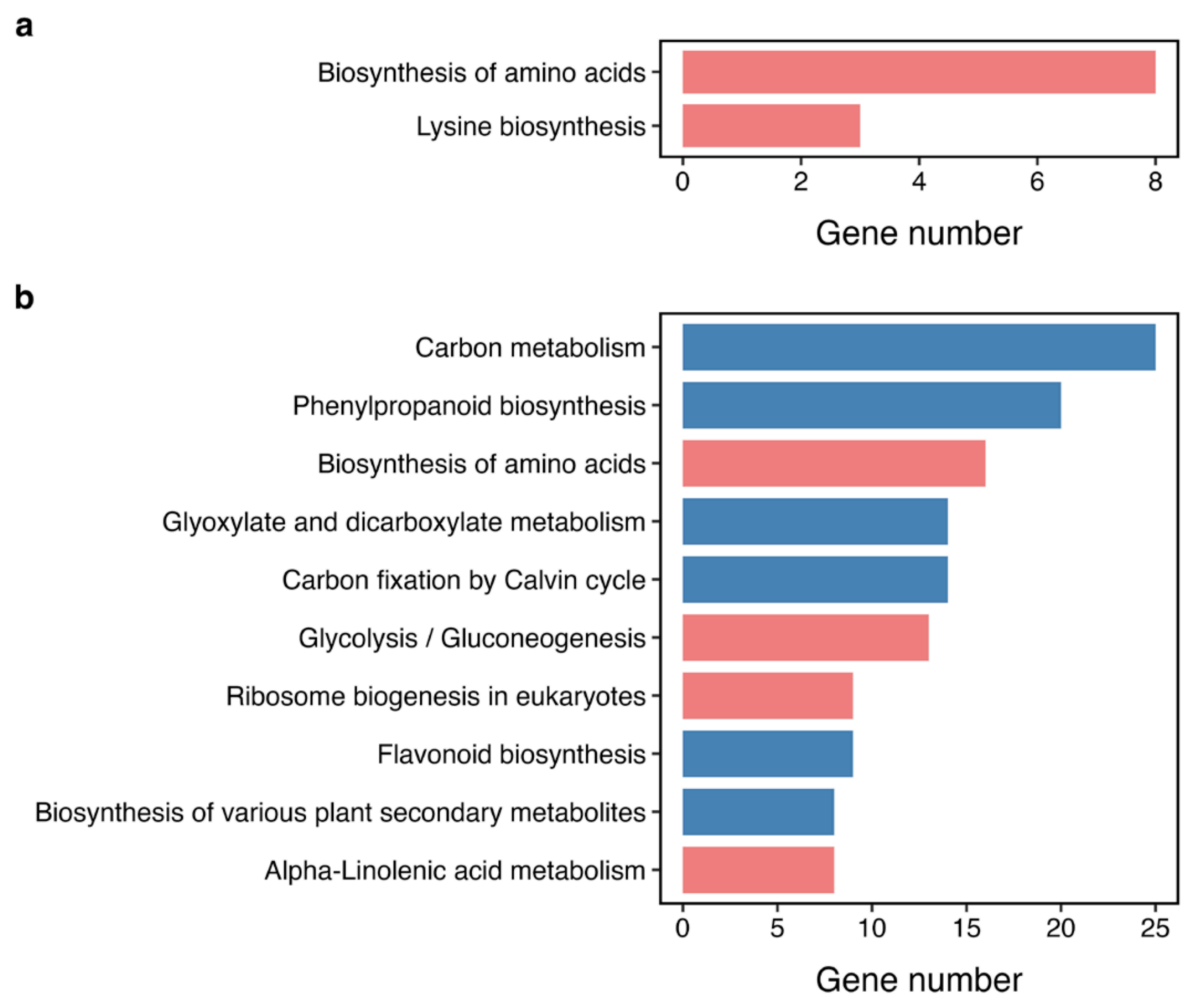
Kyoto Encyclopedia of Genes and Genomes (KEGG) pathway enrichment analysis of differentially expressed genes (DEGs) in *Spirodela polyrhiza.* The enriched pathways following *S. polyrhiza* co-cultivation with (**a**) *Terrimicrobium* sp. PS02 and (**b**) *Aeromicrobium* sp. PS05 relative to the axenic control are shown. Red and blue bars represent pathways that were significantly enriched for upregulated and downregulated genes, respectively. Only pathways meeting a significance threshold of adjusted *p*-value < 0.01 are shown.

KEGG pathway enrichment analysis further revealed distinct metabolic responses between the two bacterial isolates (Figure 7). PS02 primarily affected amino acid biosynthesis, whereas PS05 induced broader metabolic reprogramming, including the upregulation of glycolysis/gluconeogenesis and amino acid biosynthesis and the downregulation of carbon metabolism and phenylpropanoid biosynthesis.

### 3.6. Expression of Genes Associated with Starch Biosynthesis

**Figure 8:**
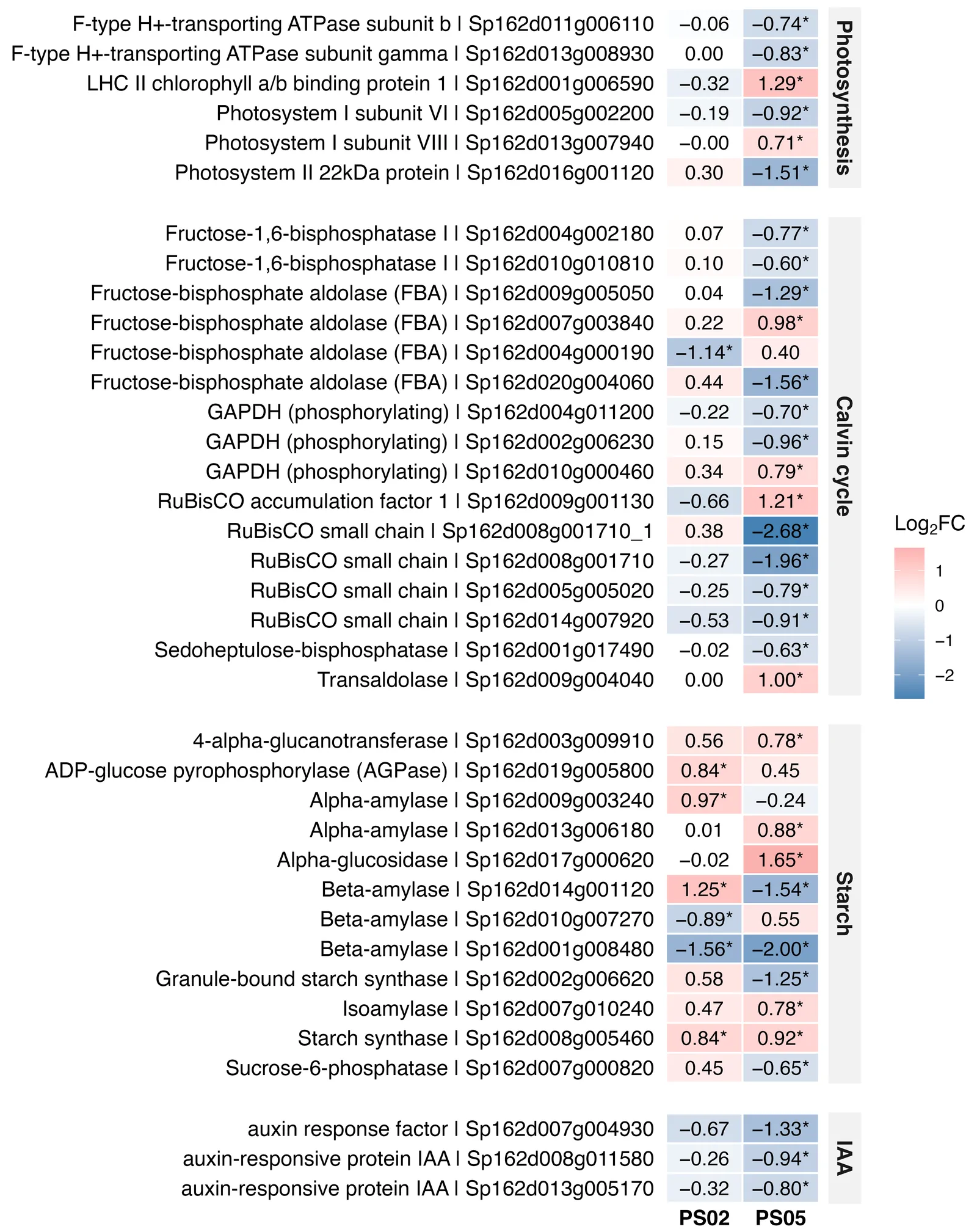
Expression profiles of key genes involved in carbon and starch metabolism in *Spirodela polyrhiza.* The heatmap displays the log_2_(fold change) of the selected differentially expressed genes following co-cultivation with *Terrimicrobium* sp. PS02 and *Aeromicrobium* sp. PS05, relative to the axenic control. Positive values indicate upregulation and negative values indicate downregulation. Asterisks denote significant differential expression (adjusted *p*-value < 0.05).

To further elucidate isolate-specific metabolic reprogramming, the expression profiles of key genes involved in photosynthesis, the Calvin cycle, and starch metabolism were extracted from transcriptomic datasets (Figure 8). Consistent with the downregulation of carbon metabolism identified by pathway enrichment analysis, co-cultivation with PS05 induced the downregulation of genes related to energy capture and carbon fixation. This includes the significant repression of multiple photosystem I and II subunits, H^+^-transporting ATPases, and core Calvin cycle enzymes, such as RuBisCO small chains, fructose-1,6-bisphosphatase, and glyceraldehyde 3-phosphate dehydrogenase. In contrast, PS02 had minor effects on photosynthetic and carbon fixation pathways. In starch metabolism, both isolates significantly altered gene expression but displayed distinct regulatory patterns consistent with net starch accumulation in the duckweed biomass. Starch synthase (Sp162d008g005460) was significantly upregulated in PS02 and PS05. Furthermore, co-cultivation with PS05 strongly repressed the genes encoding beta-amylase (Sp162d001g008480 and Sp162d014g001120), suggesting the suppression of the starch degradation pathway. PS02 upregulated ADP-glucose pyrophosphorylase (*AGPase*). These results are consistent with the enhanced starch accumulation observed in duckweed co-cultivated with *Terrimicrobium* sp. PS02 and *Aeromicrobium* sp. PS05.

**Figure 9:**
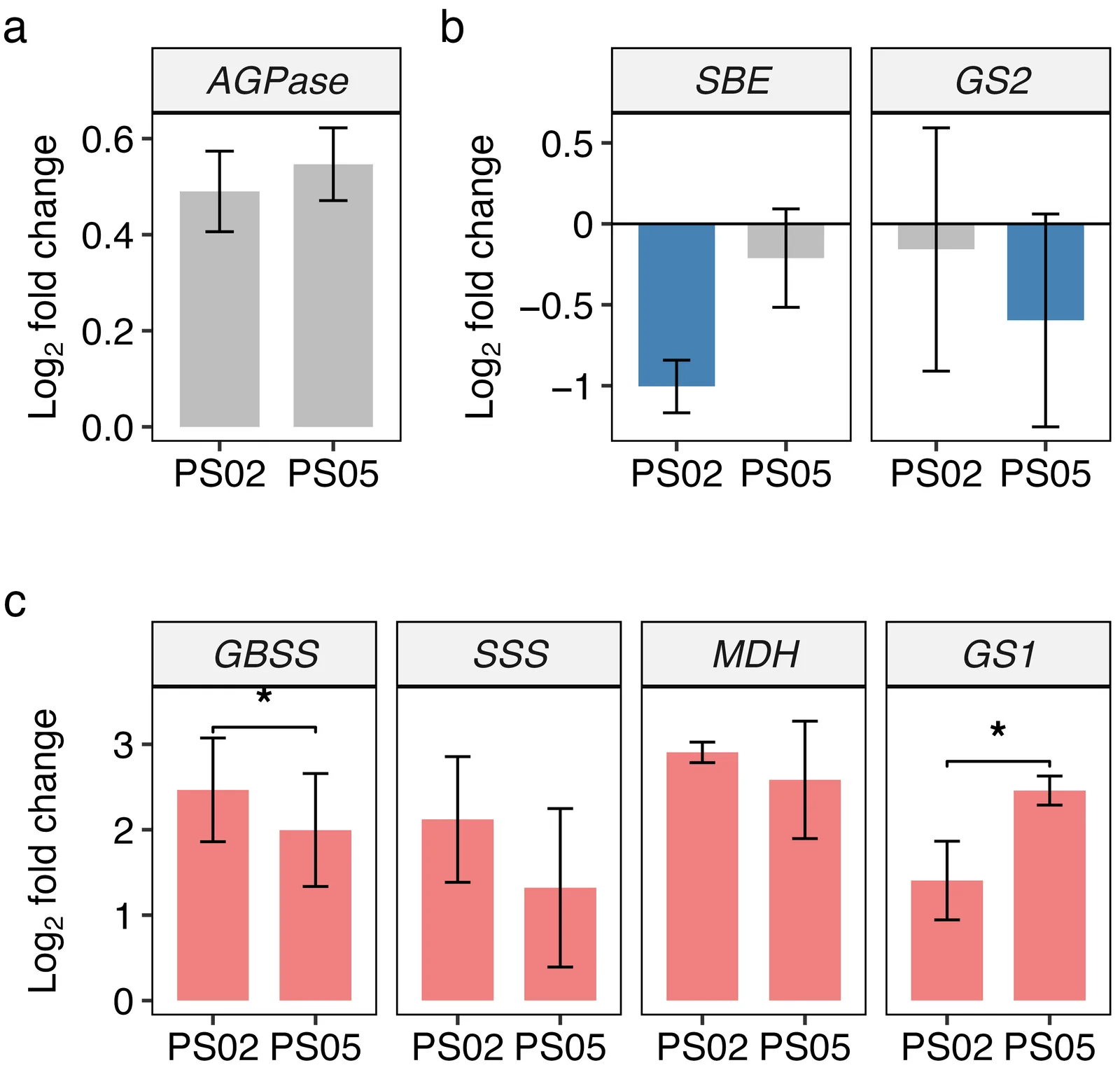
Relative expression profiles of key genes involved in starch biosynthesis and carbon metabolism. (**a**) *AGPase*; (**b**) *SBE* and *GS2*; (**c**) *GBSS*, *SSS*, *MDH*, and *GS1*. The expression levels of selected genes in *S. polyrhiza* co-cultivated with *Terrimicrobium* sp. PS02 and *Aeromicrobium* sp. PS05 were quantified using RT-qPCR. Data are reported as log_2_(fold change) relative to the axenic control. Bar colors indicate expression: red indicates upregulated expression, blue indicates downregulated expression, and gray indicates nonsignificant changes. Error bars represent the standard deviation of biological replicates, and asterisks denote significant differences in expression levels between samples inoculated with PS02 and PS05 for a given gene (Student’s *t*-test, *p* < 0.05). (**a**) *AGPase*, ADP-glucose pyrophosphorylase; (**b**) *SBE*, starch-branching enzyme; *GS2*, plastidic glutamine synthetase; (**c**) *GBSS*, granule-bound starch synthase; *SSS*, soluble starch synthase; *MDH*, NADP-malate dehydrogenase; *GS1*, cytosolic glutamine synthetase isoform 1.

The expression patterns determined using RT-qPCR were consistent with those obtained by RNA sequencing, although the magnitude of the fold changes differed between the two methods (Figure S1). Both PGPB isolates upregulated several genes involved in starch biosynthesis and carbon metabolism. Granule-bound starch synthase (*GBSS*), soluble starch synthase (*SSS*), cytosolic glutamine synthetase (*GS1*), and NADP-malate dehydrogenase (*MDH*) were consistently upregulated in both treatments compared with the axenic control (Figure 9). In contrast, starch-branching enzyme (*SBE*) was significantly downregulated in PS02-treated duckweed, whereas plastidic glutamine synthetase was specifically downregulated in PS05-treated duckweed. No significant changes were observed in the expression of *AGPase*. Overall, these expression patterns suggest that duckweed-associated PGPB alter the transcription of key genes involved in carbon partitioning and starch biosynthesis.

## 4. Discussion

### 4.1. Colonization Strategy Determines Isolate-Specific Growth Promotion

One of the key findings of this study was that the two duckweed-associated PGPB isolates established distinct colonization strategies in *S. polyrhiza*. Although *Terrimicrobium* sp. PS02 and *Aeromicrobium* sp. PS05 promoted duckweed growth, PS05 maintained bacterial populations approximately one order of magnitude higher than those of PS02 throughout the cultivation period and formed dense EPS-mediated microcolonies on the frond and root surfaces. In contrast, PS02 cells remained sparsely distributed as individual cells or small aggregates.

Successful establishment on the plant surface is widely recognized as an important factor in plant growth promotion because bacterial population size influences the local availability of phytohormones, siderophores, fixed nitrogen, vitamins, and other signaling molecules in the host plant [27,28]. Similar EPS-mediated attachment has previously been reported for duckweed-associated PGPB, including *Pelomonas* sp. MRB3 and *Acidobacteriota* TBR-22 [12,14]. The present study extends these observations by quantitatively showing that stable bacterial colonization is closely associated with enhanced plant growth. A similar trend was observed in other duckweed species, *S. polyrhiza* and *W. globosa,* where duckweed-associated PGPB also exhibited EPS production; this likely contributes directly to their high colonization and subsequent plant growth promotion [9,19]. A comparable association between EPS-mediated colonization and enhanced host growth was previously observed in the microalgal *Chlamydomonas reinhardtii*–PGPB system [29], suggesting that stable EPS-mediated colonization may represent a common mechanism underlying efficient interactions between aquatic photosynthetic organisms and their associated bacteria.

The contrasting colonization patterns observed between PS02 and PS05 may provide a mechanistic explanation for their different effects on *S. polyrhiza*. Stable, high-density bacterial attachment likely prolonged host–microbe interactions and facilitated sustained molecular communication between the bacterial cells and duckweed surface. These findings suggest that bacterial colonization is a major factor influencing the isolate-specific physiological and molecular responses in *S. polyrhiza*.

### 4.2. Extensive Transcriptomic Reprogramming Induced by PS05

Transcriptomic analyses revealed that *S. polyrhiza* responded differently to the two PGPB isolates. While PS02 altered the expression of 454 genes, PS05 induced the differential expression of 1108 genes, which was approximately 2.4-fold higher than that of PS02. PCA and Pearson correlation analyses consistently demonstrated that PS05 elicited a distinct global transcriptional profile, whereas the transcriptome of PS02-treated duckweed was considerably closer to that of the axenic control. These observations suggest that the superior growth-promoting effect of PS05 was accompanied by substantially greater host transcriptomic changes.

The magnitude of this transcriptional response closely corresponded to the contrasting colonization patterns and plant growth-promoting traits of the two bacterial isolates. PS05 is an IAA-producing bacterium that establishes stable EPS-mediated microcolonies, whereas PS02 is a non-IAA-producing and non-EPS-forming bacterium. This persistent high-density colonization may increase the local availability of bacterial signaling molecules, phytohormones, and other growth-promoting metabolites on the plant surface. In typical plant–microbe interactions, signaling molecules released by bacteria act directly or indirectly to activate plant growth and development [30], thereby potentially strengthening host–microbe communication and inducing broader molecular responses within the host. This hypothesis of localized auxin delivery is supported by the host transcriptomic response. Prolonged co-cultivation with PS05 downregulated key auxin signaling components, including auxin response factors and auxin-responsive proteins.

However, a limitation of this study was the analysis of a single transcriptomic time point at the end of the 10-day co-cultivation period. Despite the absence of early-stage temporal data, the differential regulation of the auxin signaling pathway between PGPB isolates strongly suggests active IAA-mediated signaling from EPS-forming PS05 to the duckweed host, which may contribute substantially to the extensive transcriptomic reprogramming observed in this study.

Both bacterial isolates predominantly induced transcriptional repression, with downregulated genes considerably outnumbering upregulated genes. Rather than simply activating growth-related pathways, duckweed-associated bacteria appear to extensively reprogram host metabolism through coordinated activation and suppression of multiple biological processes. This transcriptional repression likely reflects the growth-defense trade-offs in plants, wherein plants balance their growth and defense mechanisms under limited resources and energy [31,32]. This pattern suggests that bacterial inoculation stimulates plant growth directly and reprograms the overall physiological state of the host. Although the physiological and growth-promoting effects of PGPB on duckweed have been well documented [9,11,14,19], extensive transcriptomic changes in duckweed–PGPB interactions have rarely been demonstrated. Together with the contrasting colonization patterns and IAA production, these results suggest that bacterial colonization strategies and phytohormone signaling contribute to distinct host transcriptomic responses, thereby providing a molecular basis for the isolate-specific physiological differences observed in *S. polyrhiza*.

A notable characteristic of isolate PS05 was its ability to establish stable, high-density colonization through EPS-mediated microcolony formation. In the rhizosphere of terrestrial plants, the roles of EPS-producing bacteria in promoting plant growth are well-documented; EPS not only enhances colonization to drive plant–soil feedback [33] but also acts as a biostimulant capable of directly triggering host responses [34]. Similar EPS-mediated mechanisms likely drive the growth promotion observed in these aquatic plants. While the transcriptomic response in this study primarily reflects the overall duckweed–PGPB co-cultivation, such persistent colonization likely prolonged host–microbe interactions and maintained sustained exposure of the duckweed surface to bacterial growth-promoting factors, such as IAA, thereby enhancing host transcriptomic and physiological responses. Although the biochemical composition of the EPS produced by isolate PS05 remains uncharacterized, the present study clearly demonstrates its close association with stable colonization and enhanced host responses. Future studies should characterize the biochemical composition and specific biological functions of the EPS matrix.

### 4.3. PGPB Redirect Carbon Allocation Toward Starch Biosynthesis

One of the most distinctive physiological responses observed in this study was a selective increase in starch accumulation after bacterial inoculation. Both PS02 and PS05 significantly enhanced starch content without affecting protein or photosynthetic pigment content. This selective biochemical response was consistently supported by transcriptomic analyses, which showed the coordinated upregulation of genes involved in starch biosynthesis and central carbon metabolism, including *GBSS*, *SSS*, *GS1*, and *MDH*. These results suggest that the two PGPB isolates alter host carbon partitioning rather than simply promoting general biomass production.

GO and KEGG enrichment analyses supported this interpretation. PS05 preferentially enriched carbohydrate biosynthetic processes, glycolysis/gluconeogenesis, and central carbon metabolism. This coordinated regulation suggests that assimilated carbon is preferentially redirected toward starch biosynthesis rather than being uniformly distributed among multiple metabolic pathways. Therefore, the greater transcriptional response induced by PS05 was consistent with the greater starch accumulation observed in PS05-treated duckweed.

Starch accumulation in duckweed has been reported under a wide range of abiotic stresses, including nutrient starvation, unfavorable temperatures, heavy metal exposure, plant growth inhibitors, and exogenous carbon supplementation [35,36,37,38,39]. However, under these conditions, increased starch accumulation is accompanied by reduced vegetative growth because carbon is diverted from growth to storage metabolism. Consequently, although the starch concentration increased, the overall starch productivity frequently decreased.

In contrast, the present study suggests a fundamentally different physiological strategy. Bacterial inoculation simultaneously promoted vegetative growth and starch accumulation while maintaining the protein and chlorophyll contents. These observations suggest that the two PGPB isolates may redirect host carbon partitioning without imposing substantial physiological stress, thereby increasing biomass productivity and starch yield. This coordinated regulation may represent a biological mechanism distinct from conventional stress-induced starch accumulation and provide a promising strategy for improving duckweed biomass production.

### 4.4. Transcriptomic Regulation Is Associated with Starch Accumulation and Turion Formation

In addition to regulating carbon metabolism, bacterial inoculation is associated with distinct developmental responses in *S. polyrhiza*. The most conspicuous morphological change was the initiation of turion formation, which was observed exclusively in plants inoculated with either PS02 or PS05. Turions are dormant structures highly enriched in starch and are found in many duckweed species [40,41]; therefore, their formation is consistent with the enhanced starch accumulation associated with PGPB inoculation in the present study.

Transcriptomic analyses have provided additional molecular evidence associated with this developmental response. *GS1* was consistently upregulated after bacterial inoculation. Cytosolic glutamine synthetase plays an important role in nitrogen remobilization during turion differentiation and has previously been associated with starch accumulation in developing turions [42,43]. Therefore, the coordinated induction of *GS1* and visible turion formation suggests that duckweed-associated bacteria may regulate the developmental programs involved in carbon storage.

Another notable response was the increased expression of *MDH*, a key enzyme that links carbon metabolism to malate production. Although duckweed is regarded as a C3 plant, increased *MDH* expression has been reported during starch accumulation induced by plant growth regulators and other physiological stimuli [39]. The similar response observed in this study suggests that bacterial inoculation may promote alternative routes of carbon assimilation and redistribution that support enhanced starch biosynthesis.

These findings suggest that duckweed-associated bacteria influence primary metabolism and developmental processes associated with carbon storage. The simultaneous occurrence of transcriptome reprogramming, starch accumulation, and turion formation suggests that bacterial inoculation may coordinate the physiological and developmental responses in *S. polyrhiza*.

### 4.5. Proposed Mechanism Underlying Isolate-Specific Duckweed–Bacteria Interactions

**Figure 10:**
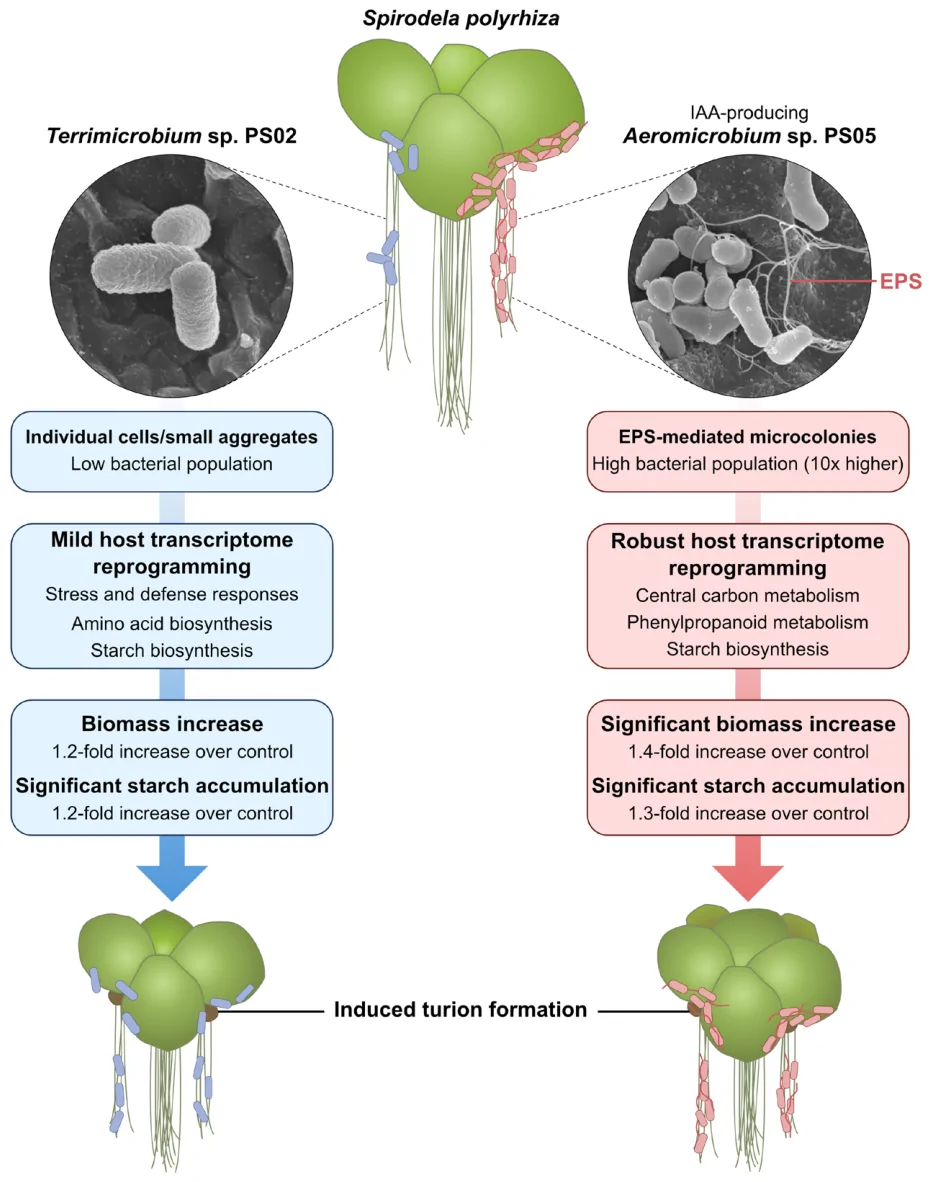
Proposed mechanistic framework describing the differential regulation of *Spirodela polyrhiza* physiology by distinct PGPB isolates. Low-density colonization by *Terrimicrobium* sp. PS02 and high-density EPS-mediated microcolony formation by *Aeromicrobium* sp. PS05 induced mild and extensive host transcriptomic reprogramming, respectively.

Based on the physiological, microscopic, biochemical, and transcriptomic evidence obtained in this study, the present study proposes a mechanistic framework describing how different PGPB isolates differentially regulate host physiology (Figure 10). The two PGPB isolates established fundamentally different colonization strategies on the surface of *S. polyrhiza*. PS05 formed stable EPS-mediated microcolonies and maintained bacterial populations approximately one order of magnitude higher than PS02. This stable colonization likely enhances host–microbe communication and prolongs host exposure to bacterial signaling molecules and growth-promoting metabolites.

Enhanced bacterial–host interactions likely induce extensive host transcriptomic reprogramming, particularly in pathways associated with central carbon metabolism and carbohydrate biosynthesis. These molecular responses may subsequently redirect host carbon partitioning toward starch biosynthesis while maintaining vegetative growth. Simultaneously, bacterial inoculation may activate developmental programs associated with carbon storage, leading to visible turion formation. These coordinated physiological and molecular responses may contribute to enhanced biomass production and increased starch accumulation.

While this study provides insights into isolate-specific duckweed–bacteria interactions, several limitations should be addressed in future research. First, because this transcriptomic analysis was conducted at a single time point (10 d of co-cultivation), future investigations should employ multiple time points to fully capture the dynamic transcriptomic shifts during initial bacterial colonization and early stages of host responses. Second, as these experiments were conducted under controlled conditions, subsequent studies should introduce these PGPB isolates to compete with the native duckweed microbiome. Finally, investigating other duckweed-associated PGPB will be required to determine whether these physiological and transcriptomic responses are conserved across different members of the duckweed holobiont. This future research will refine the mechanistic framework proposed here, ultimately providing a scientific basis for selecting beneficial microbial partners and engineering high-performance duckweed–microbiome systems for sustainable biomass production, carbon-neutral bioresource production, and future circular bioeconomy applications.

Although EPS-mediated colonization and IAA production appear to be the most plausible factors explaining the observed isolate-specific responses, additional bacterial metabolites or physiological traits may also contribute to host regulation. Future comparative studies using a broader collection of duckweed-associated PGPB isolates will be important to determine whether alternative interaction mechanisms similarly influence duckweed physiology and transcriptomic responses.

## 5. Conclusions

This study demonstrated that different duckweed-associated bacteria are associated with the differential regulation of the physiology and metabolism of *S. polyrhiza* through isolate-specific colonization strategies. *Aeromicrobium* sp. PS05 formed dense EPS-mediated microcolonies, established substantially larger bacterial populations than *Terrimicrobium* sp. PS02, and was associated with extensive host transcriptomic reprogramming. This molecular regulation appears to redirect carbon allocation toward starch biosynthesis while simultaneously promoting vegetative growth and turion formation. These findings provide new insights into duckweed–bacterial symbiosis and offer a scientific basis for microbiome engineering to improve sustainable duckweed biomass production.

## Figures and Tables

**Table 1:** Summary statistics of RNA sequencing for *S. polyrhiza* and PGPB co-cultivation.

Sample	Raw Read Count	Clean Read Count	%GC	Q30
C-1	42,414,624	42,413,142	56.74%	93.52%
C-2	43,891,642	43,890,506	57.72%	93.01%
C-3	43,627,204	43,626,516	57.15%	94.09%
PS02-1	45,452,162	45,451,716	57.51%	92.78%
PS02-2	44,388,246	44,387,244	57.70%	91.56%
PS02-3	42,538,584	42,537,884	57.60%	90.80%
PS05-1	45,512,134	45,511,914	58.54%	92.12%
PS05-2	43,647,102	43,645,722	57.10%	92.06%
PS05-3	43,699,400	43,698,616	57.84%	93.15%

C, Axenic *S. polyrhiza* control; PS02, *S. polyrhiza* co-cultivated with *Terrimicrobium* sp. PS02; and PS05, *S. polyrhiza* co-cultivated with *Aeromicrobium* sp. PS05.

**Table 2:** Gene Ontology (GO) term enrichment analysis of DEGs.

PGPB	GO Term	Description	Gene Count	Adjusted *p*-Value
PS02	GO:0019748	secondary metabolic process	10	0.001
	GO:0009808	lignin metabolic process	5	0.002
	GO:0009751	response to salicylic acid	10	0.019
	GO:0009414	response to water deprivation	14	0.019
	GO:0005991	trehalose metabolic process	4	0.023
PS05	GO:0019748	secondary metabolic process	26	<0.001
	GO:0009698	phenylpropanoid metabolic process	16	<0.001
	GO:0016051	carbohydrate biosynthetic process	24	<0.001

## Data Availability

The data presented in this study are available within the article and its Supplementary Materials. The accession numbers for the deposited RNA sequencing data and 16S rRNA gene sequences will be provided during the review process.

## Supplementary Materials

The following supporting information can be downloaded at: https://www.mdpi.com/article/10.3390/microorganisms14081850/s1, Figure S1: Comparison of RNA-seq and RT-qPCR expression data; Table S1: Taxonomic identification and plant growth-promoting traits of the bacterial isolates used in this study [44,45,46]; Table S2: Primer sequences used for RT-qPCR analysis.

## Abbreviations

The following abbreviations are used in this manuscript:

AGPase	ADP-glucose pyrophosphorylase
CFU	Colony-forming unit
DEG	Differentially expressed gene
DHM	Diluted Hutner’s medium
EPS	Extracellular polymeric substance
GBSS	Granule-bound starch synthase
GO	Gene Ontology
GS1	Cytosolic glutamine synthetase
GS2	Plastidic glutamine synthetase
HSD	Honestly significant difference
IAA	Indole-3-acetic acid
KEGG	Kyoto Encyclopedia of Genes and Genomes
MDH	NADP-malate dehydrogenase
PCA	Principal component analysis
PGPB	Plant growth-promoting bacteria
RNA-seq	RNA sequencing
RT-qPCR	Reverse transcription quantitative polymerase chain reaction
R2A	Reasoner’s 2A
SEM	Scanning electron microscopy
SSS	Soluble starch synthase
TPM	Transcripts per million
