# Supplementary material for: Isolate-Specific Modulation of Growth, Carbon Allocation, and Transcriptomic Responses in Spirodela polyrhiza by Duckweed-Associated Bacteria
Source: Microorganisms. 2026 Aug 20;14(8):1850. doi: 10.3390/microorganisms14081850 (PMC13515264; doi:10.3390/microorganisms14081850)
Supplement: Supplementary file 1 [file microorganisms-14-01850-s001.zip › microorganisms-4460088-supplementary.pdf]

Supplementary Materials

for

Isolate-specific modulation of growth, carbon allocation, and transcriptomic responses in *Spirodela polyrhiza* by duckweed-associated bacteria

Karnjana Ruenpham, Kazuhiro Mori, Yasuhiro Tanaka, Arinthip Thamchaipenet, Masaaki Morikawa, and Tadashi Toyama

Table S1 Taxonomic identification and plant growth-promoting traits of the bacterial isolates used in this study

PGPB isolates	Closest relative species (Accession no.)	Similarity (%)	Plant growth-promoting traits		
			IAA	Siderophore	Potential N_2 fixation
Terrimicrobium sp. PS02	*Terrimicrobium sacchariphilum* (NR_133878.1)	96.00	-	+	+
Aeromicrobium sp. PS05	*Aeromicrobium erythreum* (NR_024846.1)	100.00	+	+	+

Taxonomic identification of the PGPB isolates used in this study was determined via 16S rRNA BLAST analysis, showing the closest relative type strains, their accession numbers, and sequence similarity percentages. Qualitative screening of plant growth-promoting (PGP) traits was performed to detect indole-3-acetic acid (IAA) production, siderophore production, and potential nitrogen-fixing activity. Positive and negative results are indicated by (+) and (−), respectively. Briefly, bacterial production of IAA or its precursors was tested using a modified Salkowski's test [44]. Each PGPB isolate was cultured in 5 mL of R2A medium in two separate glass vials, with and without the supplementation of 0.1% (w/v) L-tryptophan, incubated at 28 °C for 48 h. The culture was centrifuged (10,000 × *g* for 5 min) to obtain the supernatant. Subsequently, 750 μL of Salkowski's reagent was added to 500 μL of the resulting supernatant and incubated at room temperature for 30 min. The development of a red-to-pink color in the L-tryptophan-supplemented tube indicated IAA production. Next, the production of siderophores was tested using a chrome azurol S (CAS) [45]. Each PGPB isolate was inoculated onto CAS agar plate (1.5% w/v) overlaid with R2A medium and incubated at 25 °C for 7 d. The development of a yellow-to-orange halo around the bacterial colony against the blue CAS-dye background indicated siderophore production. Lastly, a presumptive test for potential nitrogen-fixing activity was conducted using modified Jensen's medium [46] or modified *Azotobacter* medium (Leibniz Institute DSMZ-German Collection of Microorganisms and Cell Cultures, Braunschweig, Germany). Each PGPB isolate was inoculated onto these nitrogen-free selective media and incubated under identical conditions. The growth of a colony on either agar plate indicated potential nitrogen fixation ability.

Table S2 Primer sequences used for RT-qPCR analysis

Primer name	Gene name	Forward primer (5′-3′)	Reverse primer (5′-3′)
SpACT	*Actin* (reference)	CCAGATCATGTTCGAGACCTTCAAC	GGAGGGCGTATCCTTCGTAGATG
SSS	*SSS* (Sp162d008g005460)	TGCGACATACTCTTAATGCCCT	CCACTGGAAGACTTGCTCGTA
GBSS-1	*GBSS* (Sp162d002g006620)	TCGGTCTCCCAGAAAGCTTCAAG	GCTCTTCGGCATAATATGGGCTTAC
GS2	*GS2* (Sp162d001g006970)	CATCGCGGAGTACATTTGG	CACTTCACTGTCTTCTCCTG
AGP2	*AGP*	CAGACAGCAGAGGGTTCAGG	AGTTTAGTTCCTGCGCCTCC
GBSS-2	*GBSS*	GGAGGAGCAGAAAGGCTCAG	ACGGGGTCGAACTTGGTAAC
GS1	*GS1*	GAGCAAAATGGGAAAGGG	GCTGCTGGTGATGATATTGTG
MDH	*MDH*	GGGGCTCATCTACTCCTTC	TGGCGTCCATCTTCTTCC

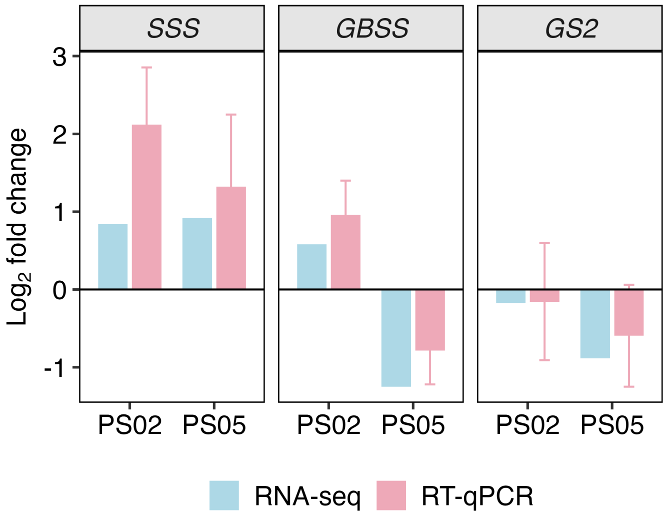

Figure S1. Comparison of RNA-sequencing and RT-qPCR analyses of selected genes involved in starch biosynthesis and carbon metabolism. The expression levels of selected genes in *Spirodela polyrhiza* co-cultivated with *Terrimicrobium* sp. PS02 and *Aeromicrobium* sp. PS05 were validated using RT-qPCR. Data are reported as $\log_2$(fold change) relative to axenic control. Blue and pink bars represent the gene expression levels determined using RNA sequencing and RT-qPCR, respectively. The analyzed genes included *SSS* (Sp162d008g005460), *GBSS* (Sp162d002g006620), and *GS2* (Sp162d001g006970). Error bars represent the standard deviations of the biological replicates.
